# 

**Published:** 1803-01-01

**Authors:** 


### MEDICAL

AND

### PHYSICAL

### JOURNAL.

CONDUCTED BY

T. BRADLEY, M.D.

R. BATTY, M.D.

AND

A. A. NOEHDEN, M.D.

VOL. IX.

FROM JANUARY TO JUNE, 1803.

171620 W.M.H. 35-B

Surgeon Gen'l's Office LIBRARY Washington, D.C. 5115

S.G.O.

*LONDON:*

PRINTED FOR

R. PHILLIPS, NO. 71, ST. PAUL'S CHURCH YARD;

AND SOLD BY ALL BOOKSELLERS IN THE UNITED KINGDOM.

[ William Thorne, Red Lion Court, Fleet Street. ]

